# Who are the chiropractic students favouring a limitless scope of practice? Exploring the relationship with personality, magical thinking, and academic achievement

**DOI:** 10.1186/s12998-022-00440-6

**Published:** 2022-07-25

**Authors:** Stanley Innes, Guillaume Goncalves, Charlotte Leboeuf-Yde

**Affiliations:** 1 College of Science, Health, Engineering and Education, Murdoch University, Perth, Australia; 2 Institut Franco Européen de Chiropraxie, 24 Boulevard Paul Vaillant Couturier, 94200 Ivry-Sur-Seine, France; 3 Faculty of Allied Health & Wellbeing, University of Central Lancashire (UClan), Preston, UK; 4 Department of Regional Health Research, University of Southern Denmark, Winsløwparken 19, 5000 Odense, Denmark

**Keywords:** Chiropractic, Chiropractic education, Spinal manipulation, Scope of practice, Magic psychology, Uncertainty, Academic success, Mass screening, Survey

## Abstract

**Introduction:**

Some chiropractors seem to have an inflated belief in the powers of spinal manipulation (SMT), for example aiming at preventing future spinal degeneration and health problems, activities that are without supporting evidence. Non-evidenced health beliefs have been shown to be associated with a tendency toward magical thinking. Holding such beliefs about SMT is associated with a limitless scope of practice (LLSoP). Recent studies have shown that “chiropractic conservatism” (ChiroCon) is also associated with such approaches. We wanted to understand ChiroCon and these attitudes toward SMT by exploring three different factors: intolerance to uncertainty, academic achievement, and tendency toward magical thinking and how they relate to ChiroCon and LLSoP.

**Method:**

A cross-sectional survey of 243 chiropractic students from an Australian chiropractic program was conducted in May 2020. Students answered a questionnaire involving a patient case-scenario for LLSoP, levels of ChiroCon, validated questionnaires on (i) Intolerance of uncertainty, (ii) Academic achievement, and (iii) Magical thinking. LLSoP was defined as wanting to treat with SMT a 5-year-old asymptomatic child for future (i) Musculoskeletal (MSK) problems and/or (ii) Non-musculoskeletal diseases. Logistic regression models were used to confirm if there was an association between ChiroCon and LLSoP and to explore associations between LLSoP and (i) Intolerance of uncertainty, (ii) Academic achievement, and (iii) Magical thinking. We repeated the same analyses using ChiroCon as the outcome variable.

**Results:**

We confirmed that chiropractic students in the more extreme ChiroCon group were more likely to want to prevent future spinal disorders in an asymptomatic 5-year-old child as compared to those with lower levels (OR = 3.9, (95%CI 1.97–7.72). This was also the case for the prevention of future diseases in the same child (OR = 6.9, (95%CI 3.11–15.06). Of the three predictor variables, magical belief was positively associated with both ChiroCon and LLSoP.

**Conclusion:**

Not surprisingly, ChiroCon is closely related to LLSoP and both were linked to magical thinking. Therefore, the questionnaire ‘Magical Health Beliefs’ could be a useful instrument to screen future chiropractic students to prevent a mismatch between student and institution. Depending on the outlook of the school, some schools would welcome these students, whereas other institutions would want to avoid them in their education program.

**Supplementary Information:**

The online version contains supplementary material available at 10.1186/s12998-022-00440-6.

## Background

In a previous survey of students at a chiropractic school, participants in general were observant of contra-indications to spinal manipulative therapy (SMT), but many seemed to consider SMT to have no or few non-indications [1]. For example, believing that chiropractic spinal adjustments can prevent degeneration of the spine and prevent disease in general. This belief was strongly associated with a positive approach to several conservative chiropractic concepts (OR 13.8 for preventing back disorders in a child and OR 20.4 for preventing disease in general), and this was confirmed later in a survey of another school, where only few students missed the non-indications to treatment but those who did were also positively inclined towards the old-time chiropractic concepts [2].

However, the use of SMT outside the musculoskeletal (MSK) field is not aligned with present scientific thinking and, further, the trust in it as a panacea is long since obsolete. Such claims would therefore be qualified as extraordinary. Perhaps these claims are correct, but extraordinary claims require extraordinary evidence, as it has been stated [3]. However, a recent systematic review on whether SMT can be used to prevent or treat non-MSK disorders has shown that SMT appears to be useful but only in methodologically weak studies, whereas, in the properly conducted studies, SMT did not come out better than sham or no treatment, regardless the condition that was treated [4]. Two recent systematic reviews did not support the concept that SMT would be beneficial on the autonomic nervous system [5, 6], neither did a more recent randomized control trial with a successful sham [7]. As for the therapeutically beneficial effect of SMT on the brain, a recent systematic review of RCTs on the subject failed to reveal any positive evidence [8]. Also, a review of all publications by one of the main proponents of the SMT-treats-the-brain concept [9] failed to find any valid positive evidence. In sum, not only could we not find any extraordinary evidence but even the ‘ordinary’ evidence appears to be lacking. Nevertheless, many chiropractic undergraduate institutions seem to adhere, at least partially, to some type of panacea model [10].

We wanted to understand why many chiropractors deviate from an MSK approach and offer treatment and cure for conditions for which they are not trained, without an obvious contemporary rationale, and, apparently, without any valid scientific evidence. We were thus curious as to the reasons why modern-day chiropractors and even chiropractic students adhere to this type of thinking, also, as we have frequently observed, when they attended or attend chiropractic schools with an MSK outlook and an evidence-based approach [2, 11].

In an attempt to unravel this mystery, we targeted three factors, namely i) an inability to cope with uncertainty, ii) academic performance, and iii) a tendency towards magical thinking to investigate their potential contributions to the adherence to both chiropractic conservatism (ChiroCon) and a limitless scope of practice (LLSoP). All three of these potential predictors have been linked with aberrant clinical practice. We further explain these variables / concepts, the relevant research, and the rationale for their choice in Additional File 1.

The aim of our study was to explore whether there are any factors that help explain the adoption of views on chiropractic conservatism (ChiroCon) in an evidence-based chiropractic education environment and its association with an increased likelihood of using SMT for conditions without an evidence-base (e.g., non-MSK). We proceeded in the following manner:

1. First, we tested if there was a link also in our study sample between conservative chiropractic beliefs (ChiroCon) and a limitless scope of practice (LLSoP), after investigating if age, sex, and year of study had an effect on any of these two outcome variables.
2. Second, to better understand the determinants of ChiroCon beliefs, we studied its association with three different factors that may help explain these: (i) Intolerance of Uncertainty, (ii) Academic ability, and (iii) Magical Thinking.
3. We, thereafter, to better understand the determinants of LLSoP, studied also its association with the same three factors.

## Methods

### Research design

A chiropractic program based at an Australian university (Murdoch University) was used for data collection. This was a cross-sectional quantitative descriptive study using an anonymous classroom handout questionnaire, as this approach facilitated the collection of a large amount of robust data in a timely and cost-effective manner.

### Ethics and participants

Ethics approval was granted by Murdoch University Human Research and Ethics (Project No 2020/022). Data were collected in a voluntary, anonymous, cross-sectional questionnaire survey between March and May of 2020. The entire chiropractic student population from the second to the fifth year of study (*N* = 320) was invited to participate via email and in-class announcement. Because of the impact of COVID-19 in Australia, the first-year students had a very different transition into the chiropractic programme, when compared to previous years, and did not have the opportunity to develop a ‘typical’ chiropractic student profile and were therefore excluded.

Students represent the future of the profession. Undergraduate education has been shown to be related to their future ability to perform basic required duties of a particular job (core tasks) as well as those extra behaviours which actively promote and strengthen the organization’s effectiveness (citizen behaviours), such as assisting others [12].

Graduated chiropractors have proven to be sensitive to research investigations into this area [13, 14]. One way to overcome this reluctance has been, instead, to investigate this phenomenon in chiropractic students, as this population, which represents the future of the profession, has been more willing to participate in such investigations [1, 2, 15, 16].

### The questionnaire / instrument

The survey contained two sections (see Additional File 1).

The first section sought the students’ age, sex, and year of study as:

i. studies on medical students have indicated that levels of intolerance of uncertainty decrease over the course of training [17]. On the other hand, there is some evidence that it may increase in the final year of chiropractic training [18].
ii. age and sex have been shown to be independent predictors of intolerance of uncertainty [19] and sex with magical thinking [20].

The second section contained the following:

i. *Intolerance of Uncertainty Scale* (IU-12): To study the intolerance of uncertainty, we used the validated 12-item version (IU-12) that utilises a 5-point Likert response ranging from ‘not at all characteristic of me’ to ‘entirely characteristic of me’ [21, 22, 23, 24, 25]. The maximum possible score is 60, reflecting high levels of intolerance of uncertainty [26, 27]. Normative data for the IU-12 were available from previous studies involving student populations [25, 28, 29].
ii. *Academic performance*: Because of confidentiality issues in obtaining academic results, students were asked if they would voluntarily self-report i) their national secondary education completion ranking score: Australian Tertiary Admission Rank (ATAR) on the understanding it would be anonymous. ATAR national ranking scores could range between 0 and 100. To validate this self-reported score, we also collected ii) their highest anatomy grade score obtained during their chiropractic education. These scores could range from Fail (below 50%) to High Distinction (above 80%). We compared this official information with self-reported data obtained from the students on their highest anatomy score. We chose this because a previous study from a chiropractic program found that students’ anatomy performance was shown to predict future academic and professional licencing examinations performance [30].
iii. *Magical thinking*: The Magical Health Beliefs (MHB) subscale is part of the Magical Beliefs about Food and Health Scale and was developed to explore a person’s intuitive attraction to suppositions regarding contagion, naturalness, certain core knowledge, as well as cognitive errors and biases [31]. Examples of such beliefs are: “Plants are living beings whose energy can be transmitted to human beings” and “The statement that red drinks improve haemoglobin is probably valid”. This validated questionnaire contains 10 items and is scored using a 5 point-Likert response format.
iv. *Clinical cases* to explore non-indications for SMT, i.e., LLSoP: For this, we included two clinical cases of a 5-year-old child based on the non-indicated use of SMT for (i) primary prevention of MSK and (ii) non-MSK conditions, using questions taken from previous studies [1, 2].
  (i) The item for prevention of MSK conditions was: “A mother wants to bring her 5-yr. old child for regular chiropractic consultations to prevent the onset of spinal disorders in the future. The child has never had back pain before. Are you willing to regularly adjust this child to avoid the onset of back disorders in the future?”. The answer possibilities were “Definitely not”/”Probably no”’/”Don’t know” and “Yes probably”/”Yes definitely” (i.e., the positive answers indicating a perception of an LLSOP).
  (ii) The item for non-MSK conditions was: “A mother wants to bring her 5-yr. old child for regular chiropractic consultations to prevent the onset of disease in the future. The case history reveals many diseases in the family (breast cancer, diabetes, lipidaemia, etc.) and the question was: “Are you willing to regularly adjust this child to avoid the onset of disease in the future?”. The answer possibilities were the same as for the MSK primary prevention case, with the same interpretation.
v. *Chiropractic Conservatism* (ChiroCon): Ten items were drawn from three previous studies relating to chiropractic conservatism (Table 1). Four items related to the concept of the ‘subluxation’[2, 32], the other six asked questions on chiropractic ‘adjustments’ [2, 18, 32]. It was previously shown that students with the higher scores were more likely to choose an inappropriate clinical approach by treating also non-indicated cases, i.e., an indication of an LLSoP [1]. A conservatism score was obtained by adding up the ‘inappropriate’ answers. Since these scores could not be considered continuous, they were further categorized by placing them in four groups ranging from low to high: group 1 (scores 0-2); group 2 (scores 3-5); group 3 (scores 6,7) and group 4 (scores 8-10). This scoring system was already used in two previous surveys [1, 2] with logical results. The construction of the items and the rationale for the determination of correct and incorrect answers have been previously explained in detail [2].

**Table 1:** The ten ChiroCon items drawn from previous studies

In your opinion, can chiropractic spinal adjustments	
Item 1	prevent disease in general?
Item 2	help the immune system?
Item 3	improve the health of infants?
Item 4	help the body function at 100% of its capacity?
Item 5	prevent degeneration of the spine?
*For each statement, choose the box that best corresponds to your opinions*	
Item 6	subluxations are the cause of all disease
Item 7	subluxations cause short-circuits of the nervous system
Item 8	subluxations can have a negative effect on the capacity of the nervous system to provide energy to tissues and organs
Item 9	it is possible to detect subluxations before symptoms appear
Item 10	it is appropriate for every person to receive chiropractic adjustments for their entire life

## Data analysis

Data were entered and analysed in SPSS v.24 (IBM Corp, Armonk NY, USA) after identifying and correcting any incomplete or corrupt data. All analyses were performed by an independent statistician (AJ). For ease of relating to the relevant analyses, all preliminary analyses are reported separately.

All survey items were allocated a dummy variable code to ensure anonymity and descriptive statistics were generated and shown in a descriptive table (Table 2). Age, U12, ATAR score and MHB were reported as the median, mean, and standard deviation. Sex and year of study were reported as frequencies. Scores on ChiroCon and LLSoP were summed and analysed as continuous data by conducting logistic regression analysis. However, we thought that this analysis was best understood as Odds Ratios, thus the scores of ChiroCon (treated both as an independent and dependent variable) were also summed and dichotomised into the categories of low scorers (groups 1 and 2) and high scorers (groups 3 and 4). The dependent variable LLSoP for each of the two scenarios (SMT preventing i) MSK and ii) non-MSK conditions in a 5- year-old child) were dichotomised in the same way. Exact numbers can be seen in Table 2.

**Table 2:** Descriptive table of demographic, independent / predictor and dependent/ outcome variables in a survey of 243 Australian chiropractic students

Variables	*N* (%)	Mean (SD); Median
Males	122 (50)	
Females	117 (48)	
Missing	4 (2)	
Age: Range 19–49		24.2 (SD 4.7); 23.00
Year of Program	*N* (Response % of year total)	
1 year	Excluded	
2 year	84 (78%)	
3 year	77 (90%)	
4 year	43 (66%)	
5 year	35 (56%)	
Missing	4	
ChiroCon score	*N* (%)	
0	19 (8)	
1	29 (12)	
2	60 (25)	
3	28 (12)	
4	27 (11)	
5	33 (14)	
6	17 (7)	
7	11 (5)	
8	7 (3)	
9	3 (1)	
10	6 (3)	
Missing	3 (1)	
ChiroCon Groups based on scores		
Group 1 (scores 0–2)	108 (44)	
Group 2 (scores 3–5)	88 (36)	
Group 3 (scores 6,7)	28 (12)	
Group 4 (scores 8–10)	16 (7)	
Missing	3 (1.2)	
Intolerance of uncertainty (IU12) *Minimum score 13, maximum 56*		30.01 (SD 8.41); 29.00
Normal score group (13–36)	170 (71.4)	
High score group (37–56)	68 (28.6)	
Missing	5	
Academic ability (ATAR) *Minimum 0—maximum 100* Missing	69	83.75 (SD 9.22); 85.00
Magical Health Beliefs (MHB) *Minimum 10 – maximum 46*		24.65 (SD 7.49); 25.00
Low group (10–25)	124 (53.7)	
High group (26–46)	107 (46.3)	
Missing	12	
Limitless Scope of Practice (LLSoP) *Prevent future spinal disorders*		
No	169 (70%)	
Yes	73 (30%)	
Missing	1	
*Prevent future diseases*		
No	207 (86%)	
Yes	34 (14%)	
Missing	2	

## Results

### Validation of data

We were concerned that students may not correctly report their entry grades into the chiropractic program, because past research has shown that chiropractic students tend to overestimate their abilities [18]. Therefore, de-identified anatomy results were obtained from the fourth year of the chiropractic program (*N* = 59) to count the frequencies of highest anatomy scores. These were then compared to the self-reported results of highest anatomy score(*N* = 42) obtained in the survey. As can be seen in Table 3, the self-reported highest anatomy scores were found to closely approximate the actual results. For this reason, we considered their reported ATAR scores also to be correct.

**Table 3:** Comparison of actual and self-reported highest anatomy scores for a single year cohort of chiropractic students

Comparison of chiropractic students’ highest anatomy score (actual vs. self-reported) in the fourth year of the program		
Grade	Actual (*N* = 59) *N* (%)	Self-reported (*N* = 42) *N* (%)
> 70	41 (70)	32 (76)
60–69	16 (27)	9 (22)
50–59	2 (3)	1 (2)
< 50	0 (0)	0 (0)

Further, the internal consistency for the survey variables (IU-12, MHB, ChiroCon) was evaluated using Cronbach’s Alpha, where a score higher than 0.7 is considered acceptable [33]. The Cronbach Alpha for scales for the three inventories were good for IU-12 (0.87) and MHB (0.85) and acceptable for ChiroCon (0.74) [34].

#### Potential interactions with age, sex, and year of study

Based on the literature, we tested if sex, age, and year of study had an effect on any of the independent variables under investigation (IU-12, Academic ATAR score, MHB, ChiroCon). This was done through the separate ANOVA analysis comparing age, sex and year of study (years 2 through 5) with the IU12, ATAR, MHB and ChiroCon. A statistically significant difference between the year group scores for ChiroCon and MHB was found (See Table 3). The scores of the final year students were significantly lower than those of the second-year students. Consequently, students’ year group was included in all logistic regression analysis involving the variables ChiroCon and MHB. (Data not shown but complete results are available on reasonable request). The groups did not have significantly different variances (Bartlett’s test = 0.28).

### Descriptive Information for all variables

In all, 243 of 320 students (75%) returned the questionnaire, of which 117 were female (49%), the mean age being 24.2 years. A description of responders is shown in Table 2 for the collected demographic and other variables under investigation.

### Confirming if there is a link between chiropractic conservative beliefs (ChiroCon) and a limitless scope of practice (LLSoP)

The more conservative group (Groups 3 and 4) found it more difficult to determine non-indications for SMT and were more willing to prevent future spinal disorders in an asymptomatic 5-year-old child than the combined Groups 1 and 2 (OR = 3.9, 95% CI 2.0–7.7).

When compared to the least conservative group, the more conservative students found it even more difficult to determine non-indications for SMT, as they wanted to prevent future diseases in a 5-year-old asymptomatic child (OR = 6.9, 95% CI 3.1–15.1).

*The association between conservative chiropractic beliefs (ChiroCon) and* *:* *(i) Magical Health Beliefs, (ii) Intolerance of Uncertainty (iii), and Academic ability (ATAR score), adjusted for Year of Program.*

Preliminary analyses, using continuous data, showed that our three potential predictors, Intolerance of Uncertainty, Academic ability, and Magical Health Beliefs, explained 27% of the variance of the ChiroCon (F(3,219) = 26.811,* p* < 0.0001, R^2^ change = 0.269) and, not surprisingly, 25% of the variance for those answering “yes” to using SMT to prevent future spinal degeneration and 8% of the variance for those answering “yes’ to using SMT to prevent future diseases (F(3.221) = 19.495,* p* < 0.001, R^2^ = 0.250 and F(3.220) = 7.21,* p* < 0.001, R^2^ = 0.077, respectively). This association was driven by Magical Health Beliefs, with the others playing smaller roles (data available from the authors on reasonable request). These links are, however, best understood as odds ratios. Further, the intervals obtained by the Likert responses do not necessarily represent equal distances, for which reason categorical analyses would be better (Table 4). The categories are shown in Table 2.

**Table 4:** Chiropractic students’ scores on ChiroCon & MHB across years 2–5 in a survey of Chiropractic Conservativism and Magical Health Beliefs

	*N*	Mean (SD)	Std. Error	95% CI
*Mean (95% CI) ChiroCon scores across years of study 2, 3, 4 and 5*				
Year 2	83	3.96 (2.56)	0.28	3.40–4.52
Year 3	76	3.05 (2.09)	0.24	2.58–3.53
Year 4	43	4.26 (2.45)	0.37	3.50–5.01
Year 5 (final)	35	2.23 (1.83)	0.31	1.60–2.86
Total	237	3.47 (2.39)	0.16	3.16–3.78
*Mean (95% CI) MHB scores across years of study 2, 3, 4 and 5*				
Year 2	81	26.44 (7.2)	0.80	24.86–28.03
Year 3	75	23.76 (7.1)	0.82	22.12–25.40
Year 4	40	26.58 (6.7)	1.06	24.43–28.72
Year 5 (final)	35	20.17 (7.2)	1.22	17.69–22.65
Total	231	24.65 (7.4)	0.49	23.69–25.60

*ChiroCon* measurement of level of chiropractic conservativism, *MHB* level of magical health beliefs

Thus, using categories rather than continuous data in the analyses, Table 5 shows that Magical Health Beliefs is the only of the three potential predictors that is significantly associated with ChiroCon, as the lower limit of the 95% CI exceeded 1 (see column 3), and the p-value was lower than 0.05 (see column 4). Students scoring high on magical health beliefs were 4.3 times more likely also to be classified as ChiroCon as compared to those scoring low (column 2).

**Table 5:** Logistic *regression analysis showing that the Magical Health Beliefs (MHB) score significantly predicts conservative beliefs (ChiroCon) about the use of spinal manipulation*

Model	Multivariate Logistic Regression		
*Variable*	ORs	95% CI	*P*
MHB (categorical)	4.3	2.0–9.1	< 0.0001
IU-12 (categorical)	1.4	0.7–2.9	0.327
Academic ATAR (continuous data)	1.0	1.0–1.1	0.101
Year of program (categorical data)	0.7	0.5–1.0	0.069

## Verification if (i) intolerance of uncertainty (IU-12), (ii) academic ability (ATAR), and (iii) magical health beliefs (MHB) adjusted for Year of program had similar links with a limitless scope of practice (LLSoP) as they had for conservative chiropractic beliefs (ChiroCon) in the previous analysis

### Preventing future i) spinal pain and ii) disease with SMT

When it came to deciding to use SMT to *prevent future spinal problems and prevent diseases in an asymptomatic 5-year-old child,* only the scores relating to magical health beliefs were found to be significant. Tables 6 and 7 are constructed identically, thus showing how, as compared to students who do not hold magical health beliefs, the believers in magic are, respectively, 2 and 4 times more likely also to believe that SMT can prevent (i) MSK and (ii) future diseases in an asymptomatic 5-yr-old.

**Table 6:** Summary of logistic regression for variables predicting the clinical decision to undertake SMT to prevent future spinal disorders in an asymptomatic 5-year-old child

Model	Multivariate Binary Logistic Regression		
*Variable*	ORs	95% CI	*P*
MHB (categorical)	2.0	1.1–3.5	0.018
IU-12 (categorical)	1.7	0.9–3.0	0.097
Academic ATAR (continuous data)	1.0	1.0–1.0	0.548
Year of program (categorical data)	0.9	0.7–1.2	0.434

**Table 7:** Summary of logistic regression for variables predicting a clinical decision to undertake SMT to prevent future diseases in an asymptomatic 5-year-old child

Model	Multivariate Binary Logistic Regression		
*Variable*	ORs	95% CI	*P*
MHB (categorical)	4.0	1.8–9.0	0.001
IU-12 (categorical)	1.7	0.8–3.6	0.172
Academic ATAR (continuous data)	1.0	1.0–1.1	0.670
Year of program (categorical data)	0.7	0.5–1.1	0.105

### Post Hoc* analysis*

Since many of the concepts of chiropractic conservatism appear to be magical, it would be possible that the adherence to the old chiropractic concepts is but a variant of magical thinking. On the other hand, we assumed that it would not be necessary for magical thinkers to adhere to the old chiropractic concepts, as there are many other aspects in chiropractic that draw on a “magical beliefs type of thinking”. A cross-tabulation of the two dichotomised variables MHB and ChiroCon confirmed that although 74% of the 43 students who scored high on ChiroCon scored high also on MHB, only 30% of the 107 who scored high on MHB scored high also on ChiroCon (Pearson Chi-Square, df (1) = 16.311, *p* =  < 0.0001).

## Discussion

### Findings

This study shows that chiropractic students with higher levels of conservative beliefs are significantly more likely to make inappropriate non-indicated clinical decisions for SMT, when compared to those with lower levels. New information is that conservative attitudes and beliefs seem to be influenced by individuals’ intrinsic factors. Thus, students who have a tendency to engage in ‘magic thinking’ are also more likely to resort to conservative thinking, and are, not surprisingly, more likely to accept for treatment also cases that are outside the scope for SMT.

However, having a closer look at the students who scored high on MHB, only 30% of them were also high on ChiroCon indicating that they could also be looking for other chiropractic-magical opportunities such as those described in the background text.

### Strong association between chiropractic conservatism and a limitless scope of practice confirmed

This study, thus, confirmed that chiropractic students with higher levels of chiropractic conservative beliefs are significantly more likely to accept a wide scope of practice. As compared to students with lower levels of conservative chiropractic beliefs, when faced with an asymptomatic 5-year-old child, they were 4 times more likely to recommend SMT to prevent future spinal disorders and 7 times more likely to believe they can prevent future diseases.

This link between ChiroCon and a LLSoP has been previously shown, both in a setting with students showing a large preference for conservative chiropractic thinking [1] and in a study with only a minority of students thinking in this way [2]. As in the present study and in the other two study settings, this type of conservative chiropractic clinical rationale meaning was not encouraged, it does not appear to be entirely the ‘fault’ of the educational institution.

### Other matters

This is the second study at this Australian university to explore conservative thinking in chiropractic students [15]. The current study had a larger percentage of responding final year students when compared to a previous study and when viewed together, it suggests that the most likely interpretation is that conservative thinking declines somewhat over the educative process. This decline is also seen in the Danish chiropractic program [2]. The pattern was also seen in magical thinking but not so for intolerance of uncertainty. This suggests that although magical thinking and intolerance of uncertainty are both attempts at dealing with the unknown, they are measuring differing constructs.

Consequently, it would appear to be overly simplistic to think that providing an evidence-based curriculum alone will inoculate students against conservative attitudes and beliefs. Not surprisingly, this is backed by the literature, as it has been shown that the Knowledge-Attitudes-Behaviour model performs poorly, when tested in real life [35].

Rather, it appears to be a matter of the much more difficult task of changing a person’s world view. This has been studied in detail and has now produced several meta-analytic and systematic reviews [36, 37] and should be considered as the basis for interventions for chiropractic educators to further research.

### Magical thinking drives this approach

This appears to be the first study to investigate the reasons for chiropractic conservative thinking and LLSoP. Magical thinking, rather than intolerance of uncertainty, or academic achievement, explained some of this confidence in the preventive powers of SMT for non-MSK conditions.

To have confidence in one’s ability to help suffering patients ‘magically’, is, in our opinion, beautifully idealistic but unfortunately unrealistic. However, since magical thinking may be an intrinsic personality trait, it is unlikely that mere education and information will change it more than marginally. In our study, as has been previously shown [2, 11], there was a small but gradual decline of magical thinking over the years of academic study (data not shown).

### Can we trust these findings?

Whether our results can be trusted would depend mainly on (i) the representativeness of the study sample and the external validity of the results, (ii) the quality of the data, and (iii) the choice of statistical analyses and the interpretation of data.

*Representativeness of the study sample:* A response rate of nearly 75% was obtained after the exclusion of the first-year cohort. No opposition to the study had been voiced among the students, so the non-response probably reflected only that all students do not attend all lectures all the time. It was not possible to sample non-responders, but a previous study has shown that there were no differences between this university-based chiropractic program and another university-based program in Australia, so these results are likely applicable to both [18] and therefore represent two of the four Council on Chiropractic Education Australasia accredited programs. It would be relevant to validate the generalizability of the findings to national and international chiropractic students and practitioners but, in our opinion, our findings have a strong face validity.

*External validity:* Previous studies in this Australian university chiropractic program have not demonstrated any significantly different attitude or personality traits, when first year students were compared to the second or third years [18, 38]. Hence, we are confident that the exclusion of the first-year cohort has not significantly impacted on our findings.

*Quality of data*: The variables of interest demonstrated acceptable levels of internal consistency as tested statistically. Students appeared to be accurately reporting their academic ability. Nevertheless, academic ability could perhaps be measured differently to confirm our findings. We chose not to create the four ChiroCon Groups, as used in previous studies, to obtain larger subgroup sizes for statistical purposes. Obviously, larger numbers will address this as well as confirm the Cronbach Alpha finding for the ChiroCon scale. Larger numbers would also allow further statical exploration (e.g., factor analyses) to address concerns raised with Cronbach Alpha such as its ability to determine uni-dimensionality [33]. Differences in findings between the study years might indicate a learning curve or may be a mere cohort effect or a combination of both. Longitudinal investigations would be needed, if anybody wants to confirm trajectories over time. Also, this would need to include the transition to practice to confirm that these findings for a student population also apply to practicing chiropractors.

*Statistical approach*: Although the authors, at this stage, have negative opinions regarding the use of SMT for non-MSK disorders, any bias this could have induced in the analyses was avoided with the assistance of an independent statistician. We made maximum use of our data by performing analyses on predictors using both continuous and categorical data and made an effort to present the data clearly with explanations to make the text understandable also for people without knowledge in statistics.

This study had a sufficient sample size for the logistic regression analyses and indicators of violation of basic assumptions were not observed.

### Other explanations

Although we consider our results trustworthy, ChiroCon would not be the only explanation of a LLSoP. Others who belong to this group would include those who will attempt to treat various non-MSK disorders via the autonomic nervous system, and the group that claim to treat the brain with SMT and other stimuli. Thus, if we take into account also, at least, these other groups, the number of chiropractors who do not keep to the MSK area becomes considerable [39]. That there is a link between magical thinking and ChiroCon was shown clearly, but all magical thinkers did not belong to the ChiroCon group.

### Perspectives in the light of the different types of chiropractic education

Although some chiropractors consider this a problem, some do not. Over the years, we have noticed that chiropractic organizations claim to be evidence-based-friendly, yet they accept pseudo-scientific approaches. An example is when the World Federation of Chiropractic (WFC) states: *"We commit to an EPIC future for chiropractic: evidence-based, people-centered, interprofessional and collaborative.*" in their declaration” Our Principles” [40]” but are not prepared to close the door on those chiropractic programs that continue to teach the old chiropractic beliefs by stating in the same document "... *and we champion the rights of chiropractors to practice according to their training and expertise* ". The latter statement may seem inoffensive to the public, but all chiropractors know that many chiropractic institutions sometimes teach, what we and others consider, highly suspect methods of diagnosis and treatment [41]. It is just part of the chiropractic ‘heritage’ and not really questioned.

Our suspicion is, therefore, that this evidence-friendly approach could be seen as paying lip service to evidence-based medicine, which seems to be specific to chiropractic, as clearly out-dated and non-evidence based ideologies would not be included in the curricula of mainstream healthcare professions and, if used in practice they would be challenged, and not, as in chiropractic, discretely accepted.

Further, the ‘overseeing’ organization, the various Councils on Chiropractic Education (owned by and run by chiropractors) have been shown to have an approach that, generally, provides a ‘big tent’, making it possible for chiropractic institutions that range from very ‘chiropractically’ conservative to not at all conservative to be ‘accredited’ as equals [41, 42]. This phenomenon is referred to as “professional diversity”, as defined in principle 12, in the same document from the WFC (36) and “respecting the past, embracing the future” is one of its strategic points, according to their Strategic Plan 2019–2022.

Thus, contrary to medicine, where hundred-year clearly outmoded practices are ousted without too much ado, in chiropractic there is obviously a strong support for, at least, some of the old concepts and the ‘you-never-know’-approach. In fact, there seems to be an unwritten requirement that the conservative groups be left alone, and that ‘respect’ is due towards them, described as ‘diversity’ [42, 43]. Thus, it appears that the LLSoP is firmly anchored in the chiropractic profession.

## Conclusions

The results from this study can therefore be of great use to both types of approaches, the MSK and the non-MSK. To prevent a mismatch between students and learning institutions we recommend that *both* those colleges that favor the old conservative approach of a limitless scope of practice or other types of magical approaches *and* institutions with a modern, MSK-only approach should screen for magical thinking to include or exclude potential students according to their requirements. Finally, the findings of this study explained less than 30% of the total variance, and this means that other factors are at play in determining clinical decisions that will require further investigation.

## Supplementary Information

**Additional file1. **Explanation of factors – IU-12, ATAR, Magical Thinking and the questionnaire

## Data Availability

Data not shown but complete results are available on reasonable request and with further Ethics approval. None.

## Abbreviations

ATAR: Australian tertiary admission rank
ChiroCon: Level of chiropractic conservativism
IU-12: Intolerance of uncertainty
LLSoP: Limitless scope of practice
MHB: Magical health beliefs
MSK: Musculoskeletal
SMT: Spinal manipulative therapy
