# Correction: Who are the chiropractic students favouring a limitless scope of practice? Exploring the relationship with personality, magical thinking, and academic achievement

**DOI:** 10.1186/s12998-022-00448-y

**Published:** 2022-09-30

**Authors:** Stanley Innes, Guillaume Goncalves, Charlotte Leboeuf-Yde

**Affiliations:** 1 College of Science, Health, Engineering and Education, Murdoch University, Perth, Australia; 2 Institut Franco Européen de Chiropraxie, Boulevard Paul Vaillant Couturier, 94200 Ivry-Sur-Seine, France; 3 Faculty of Allied Health & Wellbeing, University of Central Lancashire (UClan), Preston, UK; 4 Department of Regional Health Research, University of Southern Denmark, Winsløwparken 19, 5000 Odense, Denmark

## Correction to: Chiropractic & Manual Therapies (2022) 30:30 https://doi.org/10.1186/s12998-022-00440-6

Following the publication of the original article [1], it has been brought to our attention that several of the provided references are mismatched in the Background section and are incorrectly recorded in the Reference section.

The text in the second paragraph of the Background section originally read:

“Two recent systematic reviews did not support the concept that SMT would be beneficial on the autonomic nervous system [5, 6], neither did a more recent randomized control trial with a successful sham [7]. As for the therapeutically benefcial efect of SMT on the brain, a recent systematic review of RCTs on the subject failed to reveal any positive evidence [8]. Also, a review of all publications by one of the main proponents of the SMT-treats-the-brain concept [8] failed to fnd any valid positive evidence.”

The section should read:

“Two recent systematic reviews did not support the concept that SMT would be clearly beneficial on the autonomic nervous system [5, 6], neither did a more recent randomized control trial with a successful sham [7]. As for the therapeutically benefcial efect of SMT on the brain, a recent systematic review of RCTs on the subject failed to reveal any positive evidence [8]. Also, a review of all publications by one of the main proponents of the SMT-treats-the-brain concept [9] failed to fnd any valid positive evidence.”

The corresponding reference section originally read:

5. Picchiottino M, Honoré M, Leboeuf-Yde C, Gagey O, Cottin F, Hallman DM. The effect of a single spinal manipulation on cardiovascular autonomic activity and the relationship to pressure pain threshold: a randomized, cross-over, sham-controlled trial. Chiropr Man Therap. 2020;28(1):1–16.
6. Honoré M, Picchiottino M, Wedderkopp N, Leboeuf-Yde C, Gagey O. What is the effect of spinal manipulation on the pressure pain threshold in young, asymptomatic subjects? A randomized placebo-controlled trial, with a cross-over design. Chiropr Man Therap. 2020;28(1):1–10.
7. Meyer AL, Amorim MA, Schubert M, Schweinhardt P, Leboeuf-Yde C. Unravelling functional neurology: does spinal manipulation have an effect on the brain? A systematic literature review. Chiropr Man Therap 2019;27:60.
8. Meyer AL, Amorim MA, Schubert M, Schweinhardt P, Leboeuf-Yde C. Unravelling functional neurology: does spinal manipulation have an effect on the brain? A systematic literature review. Chiropr Man Therap 2019;27:60.

The Reference section with the alterations should be:

5. Picchiottino M, Leboeuf-Yde C, Gagey O, Hallman DM. The acute effects of joint manipulative techniques on markers of autonomic nervous system activity: a systematic review and meta-analysis of randomized sham-controlled trials. Chiropr Man Therap. 2019;27(1):1–21.
6. Araujo FX, Ferreira GE, Angellos RF, Stieven FF, Plentz RDM, Silva MF. Autonomic effects of spinal manipulative therapy: systematic review of randomized controlled trials. J Manipul Physiol Therap. 2019;42(8):623–34.
7. Picchiottino M, Honoré M, Leboeuf-Yde C, Gagey O, Cottin F, Hallman DM. The effect of a single spinal manipulation on cardiovascular autonomic activity and the relationship to pressure pain threshold: a randomized, cross-over, sham-controlled trial. Chiropr Man Therap. 2020;28(1):1–16.
8. Meyer AL, Amorim MA, Schubert M, Schweinhardt P, Leboeuf-Yde C. Unravelling functional neurology: does spinal manipulation have an effect on the brain? A systematic literature review. Chiropr Man Therap. 2019;27:60.
9. Demortier M, Leboeuf-Yde C. Unravelling Functional Neurology: an overview of all published documents by FR Carrick, including a critical review of research articles on its effect or benefit. CMT 2020;28(1):9.

The original article has been corrected.
