# Supplementary material for: Who are the chiropractic students favouring a limitless scope of practice? Exploring the relationship with personality, magical thinking, and academic achievement
Source: Chiropr Man Therap. 2022 Jul 25;30:30. doi: 10.1186/s12998-022-00440-6 (PMC9310675; doi:10.1186/s12998-022-00440-6)
Supplement: Supplementary file 1 — Additional file1. Explanation of factors – IU-12, ATAR, Magical Thinking and the questionnaire [file 12998_2022_440_MOESM1_ESM.docx]

**Rationale for variables under investigation.**

1. *Personality Intolerance of Uncertainty (IU)*

One possible contributor of simplified or stereotyped clinical approaches is personality. One such personality disposition is known as ‘intolerance of uncertainty’ (IU) and reflects a set of negative beliefs about uncertainty and represents an underlying fear of the unknown [1, 2]. Thus, IU results in a desire for predictability [2-5]. For medical practitioners, IU manifests as lower compliance with evidence-based guidelines [6] because of a tendency to unnecessarily order confirmatory diagnostic tests [7]. It is possible that higher levels of an IU drives an inflated view of spinal manipulation so that it is seen as a panacea for a complex clinical environment.

1. *Desire for predictability linked to struggles with complex thinking*

Another possibility is that this desire for certainty may not be driven by an IU. Rather it may be a reflection of difficulties in complex clinical critical thinking, either because this was never learnt or because of intellectual shortcomings [8]. If this were the case, then it would probably be reflected, at least to some degree, in lower levels of academic success. To date the possible relationship between academic success and conservative chiropractic beliefs has not been explored.

1. *Magical thinking*

The belief that spinal manipulation holds the power to benefit a wide range of non-MSK conditions, while intuitively appealing to the individual invested in training to be a chiropractor, is to this point in time, without scientific validation [9], i.e., it can be likened to magical thinking. In recent years investigators have sought to understand an individual’s tendency to adopt various unreasonable food and health ideologies or other magical beliefs which lack scientific evidence [10, 11]. High scorers in Magical Beliefs about Food and Health when compared to low scorers were shown to

- be more common in people who rely more on an intuitive thinking style rather a rational style [10].
- be more common in women [10]
- be linked with a more anxiety-based psychopathology [10]
- be more supportive of anti-vaccination views (in adult Australians) [12],
- be due to, at least in part, cognitive mistakes [13], and thus could be reflected in poor academic results
- be most influenced by intuitive thinking [11]
- manage anxiety by using this thinking style as a way of [14] avoiding issues that cause negative feelings [15]
- serve as a function for one’s self-concept by allowing people with high levels of magical thinking to identify with important reference groups and create a social identity [16].

In sum authors have concluded that adherence to such ideologies via magical beliefs gives the world meaning, clarity, and stability and thus decreases the anxiety from living in a largely uncontrolled universe [10, 11].

It is interesting to note that high levels of anti-vaccination views are also found in chiropractors described as ‘conservative’ [17] as are those who have been found to have high levels of magical beliefs. Thus, raising the possibility that magical thinking may be a contributing factor to those chiropractors who are identified as ‘conservative’.

In sum, the picture that emerges from the included variables is one of a person who is attempting to manage the complexity and uncertainty of clinical practice by creating a simpler and more ‘certain’ or predictable view of the world [18-21]. An inflated view of spinal manipulation serves this purpose by providing a panacea for a complex clinical environment that manifests in the form of clinical decisions with an increased likelihood of lower compliance with evidence-based guidelines [6]. Perhaps also the over-valuing of the spinal adjustment is part of maintaining professional self-esteem.

To date this has not been investigated.

## Questionnaire: The relationship between chiropractic student personality, beliefs and academic outcomes

**Authors:** Dr Stanley Innes, Dr Guillaume Goncalves, and Prof Niels Wedderkopp

**By completing this questionnaire, I am giving consent to become a participant in the study and that I agree to the following:**

I have read the participant information sheet, which explains the nature of the research and the possible risks. The information has been explained to me and all my questions have been satisfactorily answered. I have been given a copy of the information sheet to keep.

I am happy to be part of this study. I understand that I do not have to answer particular questions if I do not want to and that I can withdraw at any time without needing to give a reason and without consequences to myself. I also understand that once the data has been collected, it will then be anonymized, and it is no longer possible for me to withdraw.

I agree that research data from the results of the study may be published provided my name or any identifying data is not used. I have also been informed that I may not receive any direct benefits from participating in this study.

I understand that all information provided by me is treated as confidential and will not be released by the researcher to a third party unless required to do so by law.

This study has been approved by the Murdoch University Human Research Ethics Committee (Approval 2020/22). If you have any reservation or complaint about the ethical conduct of this research, and wish to talk with an independent person, you may contact Murdoch University’s Research Ethics Office (Tel. 08 9360 6677 (for overseas studies, +61 8 9360 6677) or e-mail Ethicsc@murdoch.edu.au). Any issues you raise will be treated in confidence and investigated fully, and you will be informed of the outcome.

|  | **Definitely not** | **Probably not** | | **Don’t know** | **Yes, probably** | **Yes, definitely** |
| --- | --- | --- | --- | --- | --- | --- |
| **In your opinion, can chiropractic spinal adjustments** |  |  |  |  |  |  |
| prevent disease in general? |  |  | |  |  |  |
| help the immune system? |  |  | |  |  |  |
| improve the health of infants? |  |  | |  |  |  |
| help the body function at 100% of its capacity? |  |  | |  |  |  |
| prevent degeneration of the spine? |  |  | |  |  |  |
| **We would like to know what you clinical thinking is for the following two clinical cases** | | | | | | |
| Primary prevention of back disorders  A mother wants to bring her 5-yr. old child for regular chiropractic consultations to prevent the onset of spinal disorders in the future. The child has never had back pain before. Are you willing to regularly adjust this child to avoid the onset of back disorders in the future? |  |  |  | |  |  |
| Primary prevention of diseases  A mother wants to bring her 5-yr. old child for regular chiropractic consultations to prevent the onset of disease in the future. The case history reveals many diseases in the family (breast cancer, diabetes, lipidaemia, etc.). Are you willing to regularly adjust this child to avoid the onset of disease in the future? |  |  |  | |  |  |

For each statement, choose the box that best corresponds to your opinions

|  | **Strongly disagree** | **Somewhat disagree** | **I don’t know** | **Somewhat agree** | **Strongly agree** |
| --- | --- | --- | --- | --- | --- |
| Subluxations are the cause of all disease |  |  |  |  |  |
| Subluxations cause short-circuits of the nervous system |  |  |  |  |  |
| Subluxations can have a negative effect on the capacity of the nervous system to provide energy to tissues and organs |  |  |  |  |  |
| It is possible to detect subluxations before symptoms appear |  |  |  |  |  |
| It is appropriate for every person to receive chiropractic adjustments for their entire life |  |  |  |  |  |

**Please circle the number that best corresponds to how much you agree with each item.**

|  | **Not at all characteristic of me** | **A little characteristic of me** | **Somewhat characteristic of me** | **Very characteristic of me** | **Entirely characteristic of me** |
| --- | --- | --- | --- | --- | --- |
| **1.** Unforeseen events upset me greatly. | 1 | 2 | 3 | 4 | 5 |
| **2.** It frustrates me not having all the information I need. | 1 | 2 | 3 | 4 | 5 |
| **3.** Uncertainty keeps me from living a full life. | 1 | 2 | 3 | 4 | 5 |
| **4.** One should always look ahead so as to avoid surprises. | 1 | 2 | 3 | 4 | 5 |
| **5.** A small unforeseen event can spoil everything, even with the best of planning. | 1 | 2 | 3 | 4 | 5 |
| **6.** When it’s time to act, uncertainty paralyses me. | 1 | 2 | 3 | 4 | 5 |
| **7.** When I am uncertain I can’t function very well. | 1 | 2 | 3 | 4 | 5 |
| **8.** I always want to know what the future has in store for me. | 1 | 2 | 3 | 4 | 5 |
| **9.** I can’t stand being taken by surprise. | 1 | 2 | 3 | 4 | 5 |
| **10.** The smallest doubt can stop me from acting. | 1 | 2 | 3 | 4 | 5 |
| **11.** I should be able to organize everything in advance. | 1 | 2 | 3 | 4 | 5 |
| **12.** I must get away from all uncertain situations. | 1 | 2 | 3 | 4 | 5 |

**The following sentences describe various views on keeping the body in a state of health and certain types of health care.**

|  | | **Strongly Disagree** | **Somewhat Disagree** | **Neutral;**  **no opinion** | **Somewhat Agree** | **Strongly Agree** |
| --- | --- | --- | --- | --- | --- | --- |
|  | An imbalance between energy current lies behind many illnesses |  |  |  |  |  |
|  | Colours change the organisms energy vibration in a direction that is beneficial to health |  |  |  |  |  |
|  | Plants are living beings whose energy can be transmitted to human beings |  |  |  |  |  |
|  | By massaging a diseased organs surrogate in the sole of the foot, the organ can be restored (foot reflexology) |  |  |  |  |  |
|  | An incorrect diet makes food rot in the body |  |  |  |  |  |
|  | If we don’t somehow clean our bodies, unhealthy toxins remain in them |  |  |  |  |  |
|  | It is good to detoxify one’s body every now and then with a fast |  |  |  |  |  |
|  | An illness should be treated with a medicine that has properties similar to those of the illness |  |  |  |  |  |
|  | Since our bodies are 70% water, we should be eating a diet that has an approximate water content of 70% |  |  |  |  |  |
|  | The statement that red drinks improve haemoglobin is probably valid |  |  |  |  |  |

**Demographic details.**

1. **Year of program? 1** □ **2** □ **3** □ **4** □ **5** □
2. **What is your age in years? _________**
3. **What is your sex? Female** □ **Male** □ **Identify as other** □
4. **How many hours per week are you in paid employment ? ________ Hours / week**
5. **Please write your raw ATAR score from year 12 or its equivalent? ATAR __ / 100**
6. **What was your best overall grade you achieved for an anatomy unit?**  N □ Pass □ Credit □ Distinction □ High Distinction □
7. **What was your worst overall grade you achieved for an anatomy unit?** N □ Pass □ Credit □ Distinction □ High Distinction □

**THE END. Thank you for your assistance with this survey!**

**References for rationale of included variables.**

1. Hong RY, Lee SS: **Further clarifying prospective and inhibitory intolerance of uncertainty: Factorial and construct validity of test scores from the Intolerance of Uncertainty Scale**. *Psychol Assess* 2015, **27**(2):605-620.

2. Shihata S, McEvoy PM, Mullan BA, Carleton RN: **Intolerance of uncertainty in emotional disorders: What uncertainties remain?** *J Anxiety Disord* 2016, **41**:115-124.

3. Berenbaum H, Bredemeier K, Thompson RJ: **Intolerance of uncertainty: Exploring its dimensionality and associations with need for cognitive closure, psychopathology, and personality**. *J Anxiety Disord* 2008, **22**(1):117-125.

4. Zlomke KR, Jeter KM: **Stress and worry: examining intolerance of uncertainty's moderating effect**. *Anxiety Stress Coping* 2014, **27**(2):202-215.

5. Lauriola M, Foschi R, Mosca O, Weller J: **Attitude Toward Ambiguity: Empirically Robust Factors in Self-Report Personality Scales**. *Assessment* 2015.

6. Ghosh AK: **On the challenges of using evidence-based information: the role of clinical uncertainty**. *J Lab Clin Med* 2004, **144**(2):60-64.

7. Allison JJ, Kiefe CI, Cook EF, Gerrity MS, Orav EJ, Centor R: **The association of physician attitudes about uncertainty and risk taking with resource use in a Medicare HMO**. *Med Decis Making* 1998, **18**(3):320-329.

8. **People with extremist views less able to do complex mental tasks, research suggests** [<https://www.theguardian.com/science/2021/feb/22/people-with-extremist-views-less-able-to-do-complex-mental-tasks-research-suggests?utm_term=b3d46c3212aa22cf65e8fd02a994ea54&utm_campaign=GuardianTodayUK&utm_source=esp&utm_medium=Email&CMP=GTUK_email>]

9. Cote P, Hartvigsen J, Axen I, Leboeuf-Yde C, Corso M, Shearer H, Wong J, Marchand AA, Cassidy JD, French S *et al*: **The global summit on the efficacy and effectiveness of spinal manipulative therapy for the prevention and treatment of non-musculoskeletal disorders: a systematic review of the literature**. *Chiropr Man Therap* 2021, **29**(1):8.

10. Lindeman M, Keskivaara P, Roschier M: **Assessment of Magical Beliefs about Food and Health**. *J Health Psychol* 2000, **5**(2):195-209.

11. Aarnio K, Lindeman M: **Magical food and health beliefs: a portrait of believers and functions of the beliefs**. *Appetite* 2004, **43**(1):65-74.

12. Bryden GM, Browne M, Rockloff M, Unsworth C: **Anti-vaccination and pro-CAM attitudes both reflect magical beliefs about health**. *Vaccine* 2018, **36**(9):1227-1234.

13. Gilovich TD: **The'hot hand'and other illusions of everyday life**. *The Wilson Quarterly (1976-)* 1991, **15**(2):52-59.

14. Rozin P, Lowery L, Imada S, Haidt J: **The CAD triad hypothesis: a mapping between three moral emotions (contempt, anger, disgust) and three moral codes (community, autonomy, divinity)**. *J Pers Soc Psychol* 1999, **76**(4):574.

15. Abelson RP, Prentice DA: **Beliefs as possessions: A functional perspective**. *Attitude structure and function* 1989, **3**:361-381.

16. Vyse SA: **Believing in magic: The psychology of superstition-updated edition**: Oxford University Press; 2013.

17. McGregor M, Puhl AA, Reinhart C, Injeyan HS, Soave D: **Differentiating intraprofessional attitudes toward paradigms in health care delivery among chiropractic factions: results from a randomly sampled survey**. *BMC complementary and alternative medicine* 2014, **14**:51.

18. Carleton RN: **The intolerance of uncertainty construct in the context of anxiety disorders: theoretical and practical perspectives**. *Expert Rev Neurother* 2012, **12**(8):937-947.

19. Gerrity MS, DeVellis RF, Earp JA: **Physicians' reactions to uncertainty in patient care. A new measure and new insights**. *Med Care* 1990, **28**(8):724-736.

20. Hall KH: **Reviewing intuitive decision-making and uncertainty: the implications for medical education**. *Medical education* 2002, **36**(3):216-224.

21. Jost JT, Napier JL, Thorisdottir H, Gosling SD, Palfai TP, Ostafin B: **Are needs to manage uncertainty and threat associated with political conservatism or ideological extremity?** *Pers Soc Psychol Bull* 2007, **33**(7):989-1007.
